# Bizarre Parosteal Osteochondromatous Proliferation (Nora's Lesion) of the Hand: A Report of Two Atypical Cases

**DOI:** 10.1155/2012/453560

**Published:** 2012-12-25

**Authors:** Sergi Barrera-Ochoa, Alex Lluch, Albert Gargallo-Margarit, Manuel Pérez, Roberto Vélez

**Affiliations:** ^1^Orthopaedic Surgery and Traumatology Department, Hospital Universitari Vall d'Hebron, Passeig Vall d'Hebron 119-129, 08035 Barcelona, Spain; ^2^Hand Surgery Unit, Orthopaedic Surgery and Traumatology Department, Hospital Universitari Vall d'Hebron, Spain; ^3^Hand Surgery, Institut Kaplan, Passeig de la Bonanova 9, 2n Pis, 2a Porta, 08022 Barcelona, Spain; ^4^Orthopaedic Oncology Unit, Orthopaedic Surgery and Traumatology Department, Hospital Universitari Vall d'Hebron, Spain

## Abstract

Bizarre parosteal osteochondromatous proliferation (BPOP), also called Nora's lesion, is an unusual, benign, bony lesion frequently found in the hand. Originally, two of the key radiological features used to describe such lesions were: (1) a lack of corticomedullar continuity and (2) an origin from the periosteal aspect of an intact cortex. The authors present 2 unique cases of histologically proven BPOP in which the integrity of the cortex was affected. In the first case there was medullary continuity, and in the second case there was saucerization of the underlying cortical bone. The authors support that simple X-ray evaluation is insufficient to diagnose BPOP in atypical cases. Careful axial CT scanning or MRI may prove helpful. Taking into account these new notions, histopathology gains greater importance as a diagnostic tool for this particular group of entities.

## 1. Introduction

 Nora's lesion, also called bizarre parosteal osteochondromatous proliferation (BPOP), is a rare benign osseous tumor that presents exophytic cortical growth consisting of bone, cartilage, and fibrous tissue. The most common locations are the small bones of hands and feet. Hands are four times more commonly affected than feet (1); however, lesions in other bones have been reported. It usually affects patients in their 20s or 30s (2), with no sex predilection. Fewer than 170 cases have been reported in the literature to date and its physiopathology is yet to be defined. Cartilaginous neoplasms enclose a wide variety of lesions with varying clinicopathologic behavior, and Nora's lesion can be easily misdiagnosed. This benign lesion of the bone might be mistaken for a malignant process because of its high rate of local recurrence (20–55%), the potential for rapid growth, and its atypical histological appearance.

 Through a literature review, we present two cases of BPOP, to illustrate the clinical, radiographic, and histologic features.

 We report two new cases with an unusual radiographic feature consisting of cortical destruction. Furthermore, case 1 is unique for its medullary invasion after a first surgical intervention. The absence of such continuity has been singled out as a critical imaging feature of BPOP. This case may indicate that this is, in fact, not a reliable method for either identifying or excluding BPOP.

## 2. Case Report 1

**Figure 1:**
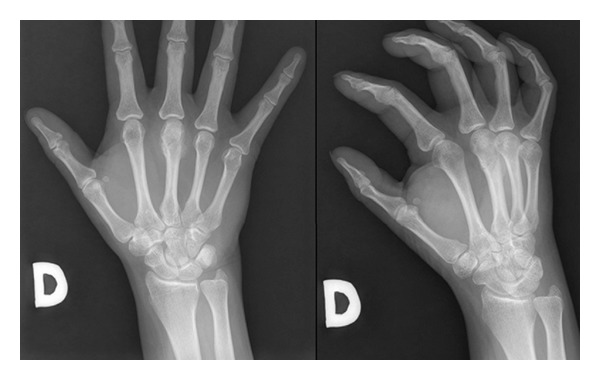
Plain radiograph of the right hand showing a dense mass extending from the dorsal aspect of the third distal metacarpal.

**Figure 2:**
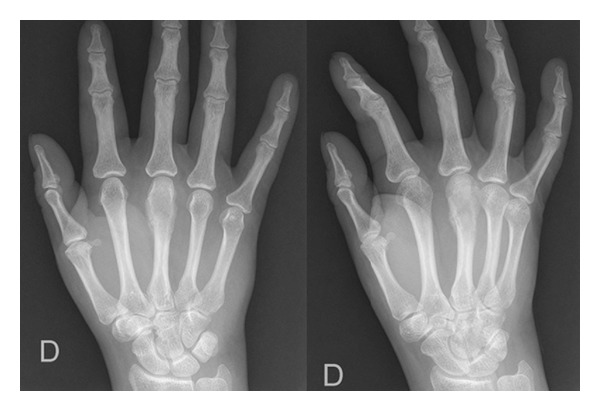
Radiograph taken 3 months postoperatively indicating that the patient had local recurrence.

**Figure 3:**
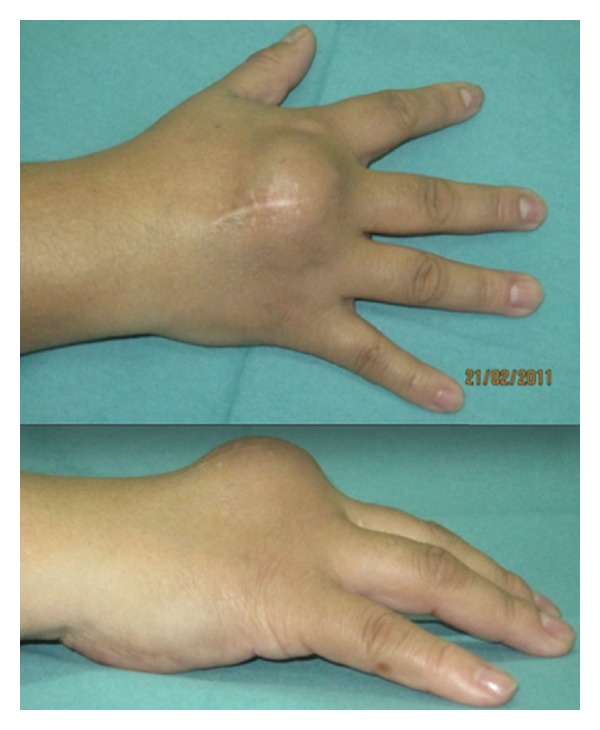
Clinical presentation of the local tumor recurrence in the dorsal hand.

**Figure 4:**
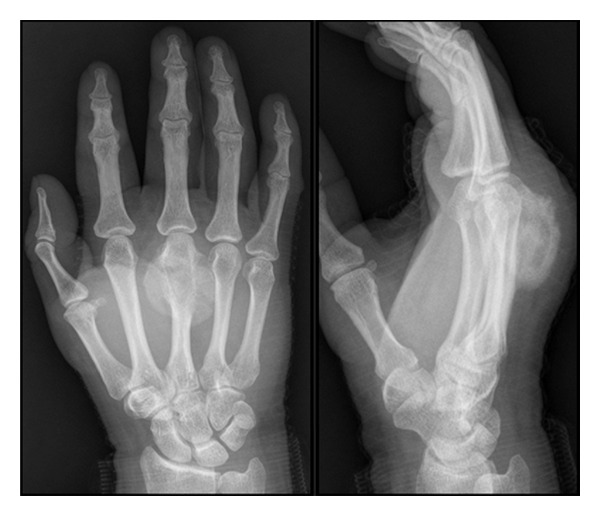
Radiograph taken preoperatively showing rapid growth of the dorsal mass.

**Figure 5:**
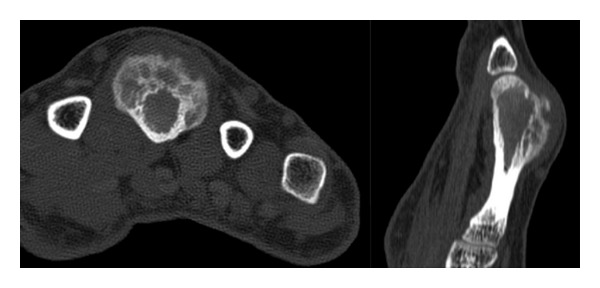
CT axial and sagittal images of the third metacarpal demonstrating a small focal area of continuity between the medullary cavity of the lesion and that of the underlying bone.

**Figure 6:**
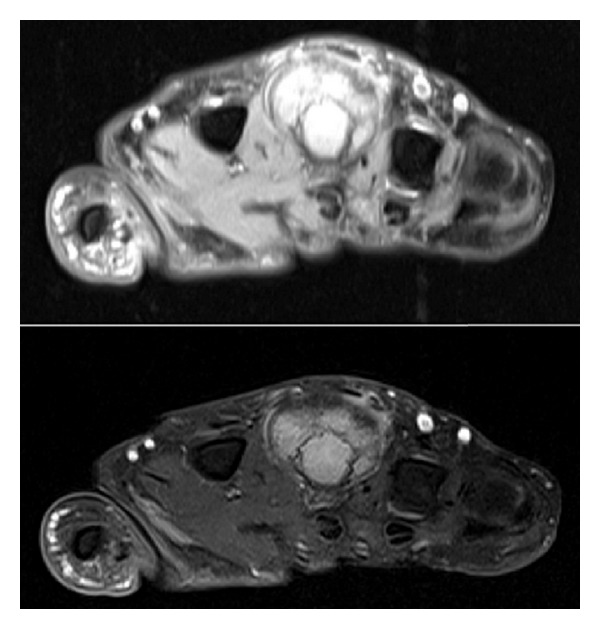
Axial T1/T2-weighted postoperative MR images of the third metacarpal demonstrate a somewhat mushroom-shaped, pedunculated lesion arising from the dorsal aspect of the third metacarpal with an internal signal similar to that of the underlying marrow.

 A 39-year-old woman was referred to our hospital after undergoing resection of a surface-based lesion involving the dorsal aspect of her right third metacarpal (Figure 1), which had been diagnosed as osteochondroma. The dorsal mass recurred with rapid growth (Figure 2) causing her pain, increased swelling, and progressive limitation of range of motion. There was no history of trauma. On physical exam, she was noted to have a well-healed dorsal incision overlying a prominent, firm mass in the distal-most aspect of the third metacarpal (Figure 3). Her metacarpophalangeal range of motion was 0°/40° and had no neurovascular lesions. A radiograph showed an ossified mass bordering the dorsal aspect of the third metacarpal (Figure 4). On the computed tomography (CT) exam, there was evident continuity between the medullary cavity of the lesion and that of the underlying bone (Figure 5). Magnetic resonance imaging (MRI) revealed a mass arising from the dorsal surface of the third metacarpal that resembled a focus of mature ossification with similar medullary and cortical components (Figure 6). 

**Figure 7:**
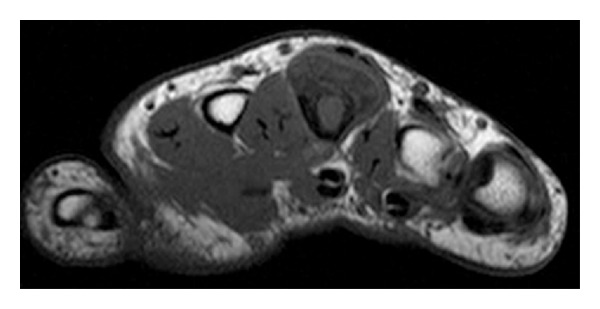
On an axial T1-weighted preoperative MR image of the area, the lesion is composed predominantly of central signal isointense to that of the normal bone marrow with a thin low signal periphery consistent with the cortical bone. Direct continuity with the underlying marrow of the bone is not noted.

Imaging prior to her initial resection was reviewed. Plain films showed an expansive lesion over the dorsal aspect of the distal third metacarpal with no medullary continuity with the lesion. MRI prior to the initial resection revealed a lesion with a corticated rim and a cartilaginous cap (Figure 7).

 The differential diagnosis at this point included osteochondroma, periosteal osteosarcoma, and periosteal chondrosarcoma.

 The mass was core biopsied and histopathology revealed proliferative and irregular osseous-cartilaginous interfaces with occasional bizarre nuclei, suggestive of BPOP.

**Figure 8:**
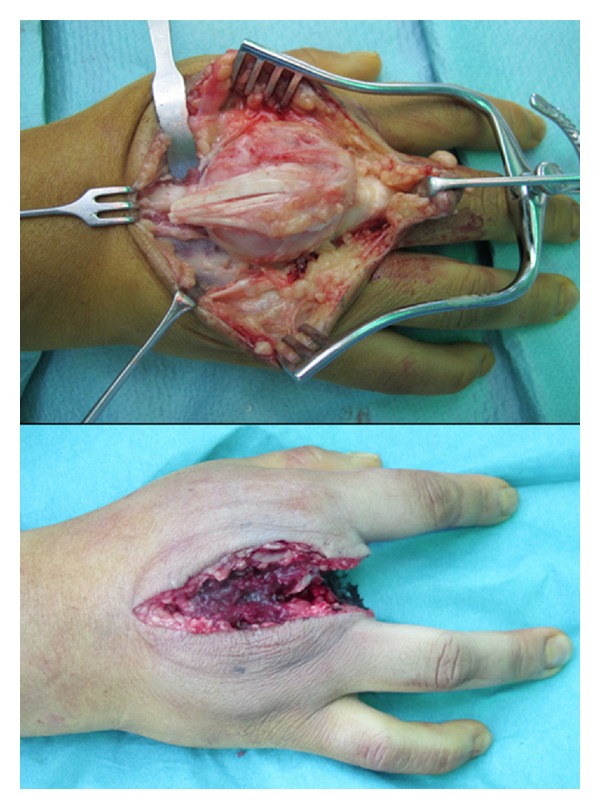
Dorsal longitudinal approach to the third metacarpal showing the dorsal tumor. Amputation of the third metacarpal.

 Due to the recurrent aggressive pattern, the patient underwent amputation of his right third ray in a standard fashion ten months after the second resection (Figure 8).

**Figure 9:**
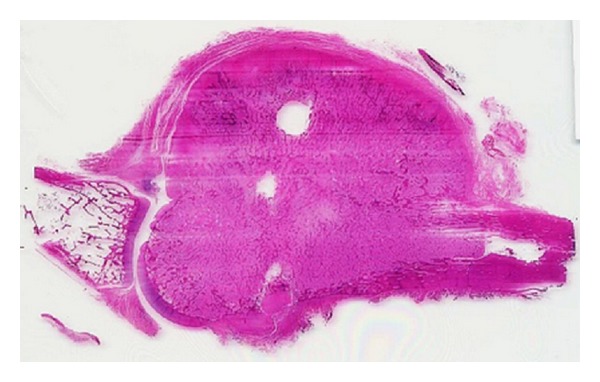
A whole mount of the lesional tissue showing the direct communication of the lesion with the host bone.

**Figure 10:**
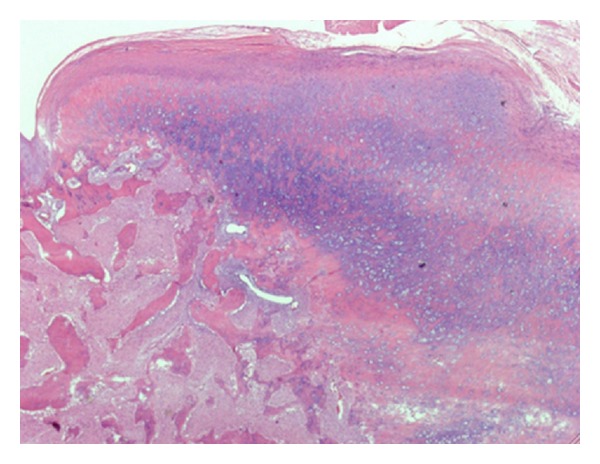
Low-power view of the lesion, demonstrating a cartilaginous cap and bony spicules (hematoxylin-eosin stain; magnification ×4).

 The bony mass measured 2.7 × 2.2 × 2.0 cm. Histopathology revealed a definite area of communication between the metacarpi and the lesion (Figure 9). Despite the radiological findings, the histopathologic examination (Figure 10) confirmed the diagnosis of BPOP.

**Figure 11:**
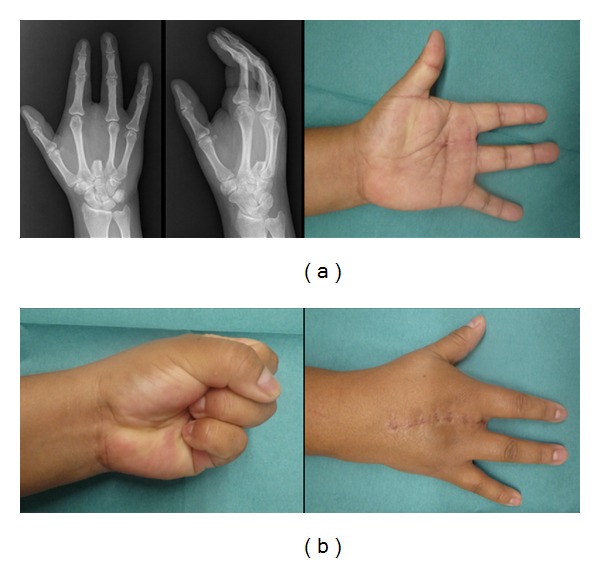
Eighteen-month postoperative clinical and radiological presentation demonstrating full range of motion without tumor recurrence.

 No complications were observed postoperatively and the patient remained disease-free at the two-year followup. The patient had a full range of motion of the remaining digits of the right hand and was able to perform all activities of daily living (Figure 11).

## 3. Case Report 2

**Figure 12:**
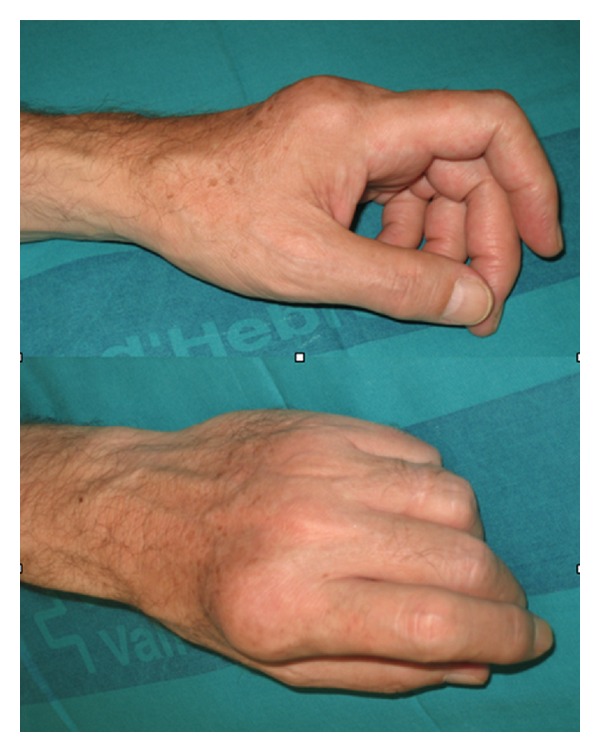
Clinical presentation of the dorsal mass over the radial aspect of the distal second metacarpal.

 The patient was a 54-year-old right-hand-dominant male who presented to us with a fast growing mass in his left hand. He had no history of trauma or surgery. On physical examination of his left hand, a dorsal firm mass over the radial aspect of the distal second metacarpal was detected (Figure 12). He had normal range of motion and intact neurovascular function. 

**Figure 13:**
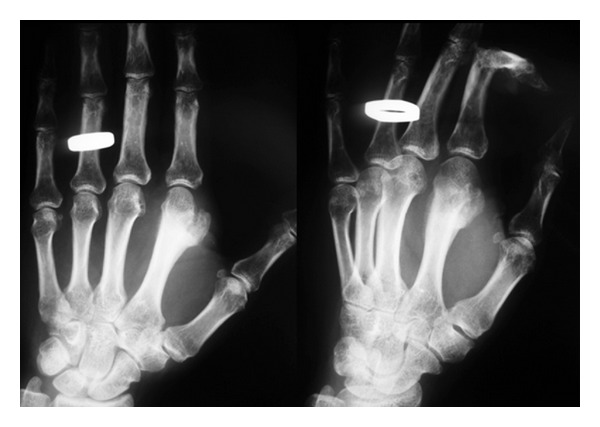
Case  2: an AP and oblique radiographs of the hand demonstrating a somewhat pedunculated mass arising from the lateral surface of the second distal metacarpal and resembling a focus of mature ossification with distinct medullary and cortical components.

**Figure 14:**
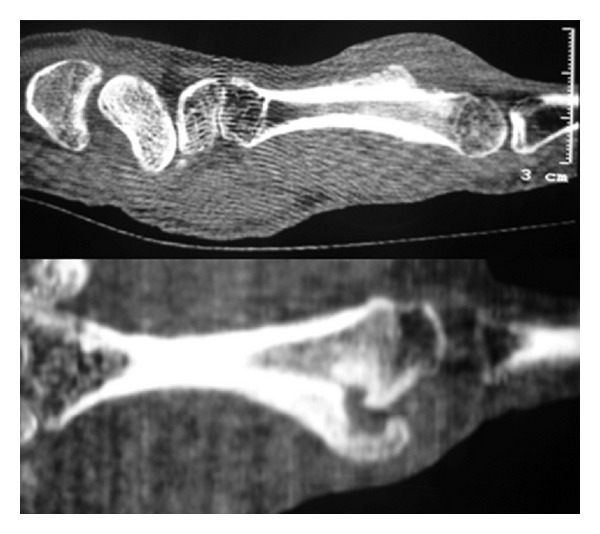
CT sagittal images of the second metacarpal demonstrating saucerization of the underlying cortical bone.

**Figure 15:**
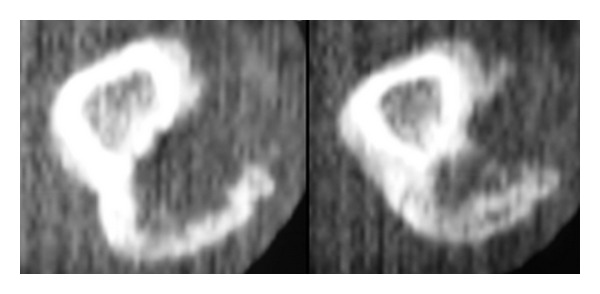
Careful axial CT scanning of the second metacarpal confirming there was not an area of continuity between the medullary cavity of the lesion and that of the underlying bone.

 Radiographic examination revealed a 2.0 × 1.0 × 1.8 cm. focal ossified lesion with a chondroid matrix (Figure 13). The CT image demonstrated saucerization of the underlying cortical bone (Figure 14). There was no continuity between the medullary cavity of the lesion and that of the underlying bone (Figure 15).

 The suspected diagnosis at this point included periosteal chondroma, periosteal osteosarcoma, periosteal chondrosarcoma, and BPOP.

**Figure 16:**
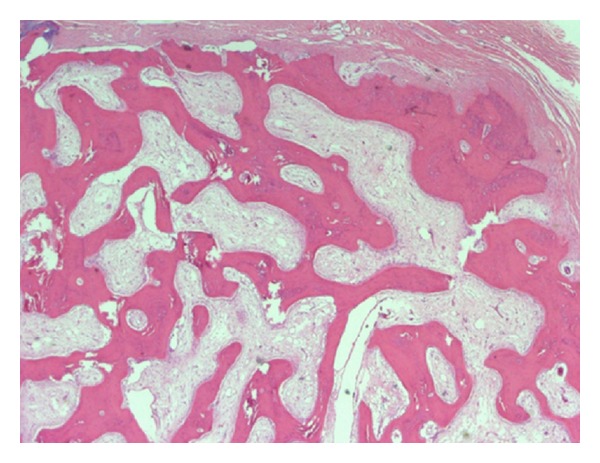
Medium-power view of bony spicules with intermixed cartilage and intraosseous fibrous tissue (hematoxylin-eosin stain; magnification ×10). Osteoblastic activity is prominent; osteoclastic activity is also appreciated.

**Figure 17:**
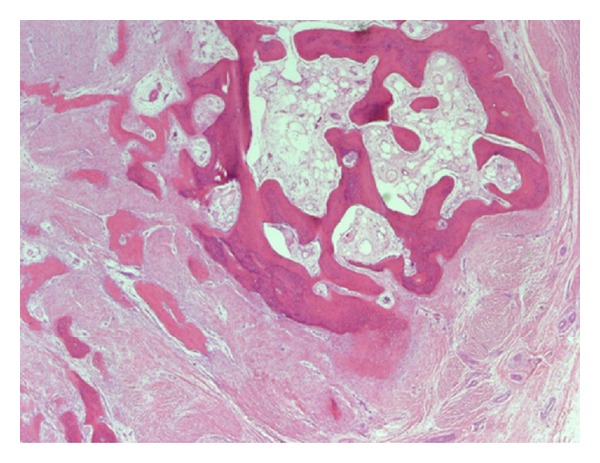
Light micrograph of the osteocartilaginous interface of the lesion showing disorganized cartilage with irregular ossification (hematoxylin and eosin stain; original magnification ×200).

The mass was core biopsied and histopathology was the suggestive of BPOP (Figures 16 and 17).

 After considering the treatment options, the patient elected to proceed with surgical excision. Surgery revealed an osteochondromatous lesion on the surface of the distal second metacarpi that did not communicate with the medullary canal. There was a shallow depression in the underlying cortex, correlating with the radiographic findings. The lesion was isolated and removed with osteotomes and a rongeur, and the underlying periosteum was excised to prevent recurrence.

 The specimen measured 2.0 × 1.4 × 2.0 cm. Again, despite the radiological findings, the histopathologic examination confirmed the diagnosis of BPOP.

**Figure 18:**
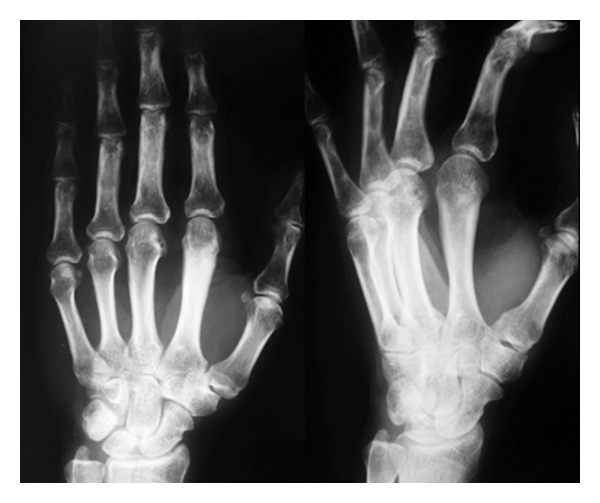
Two-year postoperative radiological presentation without tumor recurrence.

 At his four-year followup, he had no evidence of tumor recurrence (Figure 18). Hismetacarpophalangeal joint range of motion was complete. He had no complaints of pain.

## 4. Discussion

 Cartilaginous neoplasms of the musculoskeletal system represent a wide variety of lesions with varying clinicopathologic behaviors. BPOP is a benign but locally aggressive fibroosseous mass that has striking clinical similarities with osteochondroma [1] and periosteal chondroma [2]. There are unresolved issues about this rare disease regarding its etiology, diagnosis, and treatment.

 Clinical history and physical examination alone are not sufficient to reach a diagnosis. While BPOP is most common in the fourth decade, osteochondromas and periosteal chondroma are more prevalent in the second and third decades. Pain is not a helpful discriminator, as it may or may not be present. In addition to their similar clinical presentation, these tumors can also have similar radiographic and histologic appearances.

 All lesions exhibit a well-demarcated ossified mass in a juxtacortical position, with or without sclerotic borders. The key radiographic feature is the cortical aspect of the affected bone and its continuity (or lack of) between the lesion and the underlying medullary cavity. BPOP and periosteal chondromas normally do not have continuity with the medullary cavity, in contrast to osteochondromas. BPOP originates from the periosteal aspect of an intact cortex, whereas the periosteal chondroma exhibits a characteristic saucerization of the underlying cortex.

 X-rays alone are sufficient to diagnose BPOP in typical radiographic appearance and typical clinical findings [3, 4]. In the two atypical cases we have presented, plain radiographs were not conclusive in determining the presence or absence of medullary continuity. Careful axial CT scanning or MRI can be helpful when radiographs are inconclusive. Nonetheless, medullary continuity or cortical integrity should not be considered as discriminating factors, as we have seen in these two cases. 

 Currently, this concept is under discussion due to the recent appearance of BPOP cases in continuity with the medullary cavity after an event of trauma, agreed with authors as Rybak et al. have published in skeletal radiology regarding atypical radiology in Nora [5]. Some authors suggest that florid reactive periostitis, BPOP, and turret osteochondroma may reflect points along the same continuum with trauma as the likely inciting event [4]. On the other hand, there are other hypotheses about the etiology of BPOP, against the history of trauma or lesion progression [6].

 This challenges the concepts that the presence of medullary continuity will always distinguish osteochondromas from BPOP (case 1) and that BPOP always originates from an intact cortex (case 2) and raises the possibility that relying solely on these criterions may lead to misdiagnosis.

 Taking these concepts into account, histopathology becomes an even more important diagnostic tool for this group of entities. 

 Due to the similarities between the discussed entities, BPOP lesions can be easily misdiagnosed. Differentiating between these lesions is important as BPOP often requires more extensive surgical resection and has a higher recurrence rate compared with the rest. Surgical excision is the treatment of choice for BPOP lesions. With such treatment, the recurrence rate is high at 50% to 55%. A key feature to the preoperative planning is being prepared to reconstruct either the bone or ligaments in order to achieve the required safety margin. Michelsen et al. [7] described that excising the underlying periosteal tissue and any suspicious-looking cortex has been shown to be beneficial in preventing recurrence. We recommend careful resection of the underlying periosteum and cortex, because as in our case, it may be the cause of intramedullary involvement in the recurrence. Even in cases of recurrence, repeating local excision is advocated, rather than aggressive surgery [8]. In our case, due to the tumor's aggressive behavior, amputation was chosen for progression of the lesion [9].

 Due to local recurrence rates and a lack of adjuvant therapy options, the Nora lesion will continue to pose a challenge for orthopedic surgeons. Additionally, at the present time, there is no standardized screening protocol or follow-up regimen given its rarity. Therefore, treatment and follow-up care should take place in specialized centers.
